# Role of minimally invasive surgery in paediatric pulmonary metastatic disease

**DOI:** 10.3332/ecancer.2025.2026

**Published:** 2025-11-13

**Authors:** Timothy B Lautz, Rodrigo Chaves Ribeiro

**Affiliations:** 1 Division of Paediatric Surgery, Ann & Robert H Lurie Children’s Hospital of Chicago, Chicago, IL 60611, USA; 2 Department of Paediatric Surgery, Children’s Cancer Hospital, Barretos Cancer Hospital, Barretos 14784058, Brazil

**Keywords:** children, thoracoscopy, thoracotomy, pulmonary metastasectomy

## Abstract

The role of minimally invasive surgery (MIS) in paediatric pulmonary metastasectomy is evolving, reflecting advances in imaging, localisation and instrumentation. Compared with thoracotomy, thoracoscopy offers benefits of reduced postoperative pain, shorter recovery and easier reoperation. However, limitations include anaesthetic challenges in smaller children and lack of manual palpation, which may miss subpleural nodules, which is particularly important in chemoresistant tumours such as osteosarcoma and nonrhabdomyosarcoma soft tissue sarcoma. MIS is most suitable for limited disease, peripheral nodules and histologies where complete manual exploration is unnecessary. Indications depend on tumour type, number and location of lesions, as well as patient stability and institutional expertise. Advances in nodule localization—such as wire or coil marking, fluorescence imaging and radiotracers—have improved thoracoscopic precision. Wedge resection remains preferred for peripheral nodules, with anatomic resection reserved for central or larger lesions. MIS contraindications include extensive disease, inability to tolerate single-lung ventilation or lack of required resources. Optimal outcomes depend on experienced multidisciplinary teams and readiness to convert to open surgery when needed. Overall, thoracoscopy is a safe, effective option in selected paediatric patients, providing therapeutic benefit while minimising morbidity when applied judiciously to tumour biology and disease extent.

## Introduction

The role of a minimally invasive approach to lung metastases in children is in evolution. Minimally invasive surgery (MIS) can offer significant benefits, including reduced postoperative pain and quicker recovery when compared to open surgery with thoracotomy. In addition, reoperation after thoracoscopy is significantly less complicated than after thoracotomy. After thoracotomy, there may be a fusion of the intercostal space, which complicates repeat access, often increases adhesions, and can contribute to future scoliosis. However, thoracoscopy also has its limitations, particularly in smaller children, including anaesthetic challenges, equipment requirements, and importantly, an inability to palpate the lung parenchyma. In current practice, thoracoscopy is the preferred approach when there is limited disease burden, all nodules are peripheral or a lobectomy is planned in patients with tumour types that do not require comprehensive palpation of all lung surfaces. Additional applications remain under investigation.

## MIS indications

Pulmonary metastasectomy (PM) is generally indicated for patients who have had successful local control and have residual pulmonary metastases after induction chemotherapy. Pulmonary metastatic response to chemotherapy is assessed with computed tomography (CT). For tumours that are generally responsive to chemotherapy and/or radiotherapy (RT), including Wilms tumour, Ewing sarcoma, germ cell tumour, rhabdomyosarcoma and hepatoblastoma, the role of PM is fairly limited. For these tumours, metastases will often resolve with initial chemotherapy. Whole lung RT is the first-line therapy for residual pulmonary disease in Wilms tumour, Ewing sarcoma, rhabdomyosarcoma and other radiosensitive tumours. However, for tumours that are poorly responsive to chemotherapy and RT, including osteosarcoma, nonrhabdomyosarcoma soft tissue sarcomas (NRSTS) and adrenocortical tumour, PM plays a much more critical therapeutic role.

For patients who will benefit from PM, the use of a MIS approach (i.e., thoracoscopy) is influenced by the tumour type, available resources, as well as the size, number and location of the nodules. Thoracoscopy is also dependent on the patient’s hemodynamic stability and ability to tolerate one-lung ventilation.

- Tumour type: For some tumours, including osteosarcoma and NRSTS, which are relatively resistant to chemotherapy and radiation, aggressive attempts to remove even tiny nodules below the detection sensitivity of CT may be important. Multiple studies have shown that thoracotomy with palpation of the lung can identify lesions not detected on pre-operative CT in 20%–25% of patients with osteosarcoma. Without manual palpation, the detection of these occult nodules is not possible with thoracoscopy. Despite this evidence that open surgery often allows for the detection and removal of viable nodules not detected on preoperative imaging, this has never been shown to produce a survival benefit. Therefore, while thoracoscopy is used less frequently for osteosarcoma and NRSTS compared to other tumour types, the MIS surgical approach is perfectly acceptable in select clinical scenarios. In fact, patients with osteosarcoma and oligometastatic pulmonary metastatic disease are currently eligible for a phase III randomised trial in the Children’s Oncology Group comparing outcomes with thoracoscopy versus thoracotomy (AOST2031).
- Nodule number: In general, thoracoscopy is reserved for patients with no more than 3–4 lesions per side. This is driven both by limitations in the ability to detect and remove more than a few nodules, as well as considerations related to the impact on pulmonary function. MIS stapled wedge resections generally require the removal of a larger cuff of normal lung tissue compared to open wedge resections. While well tolerated for a few resections, a larger number of MIS wedges can have negative implications on pulmonary reserve due to the removal of excessive normal lung.
- Nodule location and size: MIS approach for wedge resection, lobectomy and segmentectomy are all safe and feasible. Wedge resection is generally reserved for peripheral nodules, <3 cm in size. Larger and/or central nodules (located <2 cm from the bronchus or vessels) are best treated with segmentectomies or lobectomies, which can still be performed by MIS, depending on the team's experience. When tumour involves multiple lobes of the lung and includes one or more central nodules, anatomic lobectomy or segmentectomy may not be feasible. In these cases, thoracotomy may allow for an enucleation procedure that is not possible with thoracoscopy.

## MIS contraindications

Contraindications for MIS PM include both patient factors and available resources.

- Patient factors: MIS is contraindicated for patients presenting with more extensive pulmonary disease, hemodynamic instability or pulmonary insufficiency, limiting the ability to tolerate one lung ventilation. While long-term survival in children with extensive pulmonary disease is rare, surgery may prolong survival and produce some long-term cures. However, an open approach to surgery in these more extreme scenarios is recommended.
- Available resources: Successful MIS PM relies on the availability of appropriate resources, as well as the experience of the surgeon, anaesthesiologist and other team members. While larger nodules located very close to the pleural surface can often be identified by standard white light imaging and palpation, smaller and (even slightly) deeper nodules, often benefit from localisation. Adequate localisation can reduce the potential need for conversion to open surgery to facilitate nodule identification. A variety of localisation techniques, ranging from wire or coil placement under CT-guidance, fluorescence-guided surgery or radiotracers are possible, and the choice of technique is based on surgeon and institutional preference. Nonetheless, the availability of one or more reliable localisation techniques is essential.

Other resource considerations include the availability of standard MIS equipment, near-infrared imaging equipment if fluorescence-guided surgery is employed for nodule localisation, and intra-operative fluoroscopy if microcoils or fiducials are used for localisation.

Finally, experience with and equipment for one-lung ventilation are essential.

## Surgical approach

### Patient position

The choice of surgical approach depends on lesion characteristics: site, size and involvement of one or both lungs. The patient's position must allow for the greatest access to the areas of interest and uses gravity to aid in keeping the uninvolved lung or other tissue out of the field of view. The procedure can be done in the lateral position and in specific cases in the supine or prone position. The lateral position provides an excellent visualisation and access to all surfaces of the lung. For lateral position, an axillary roll may assist in positioning. The surgical team stands in front of the patient for an anterior approach. The monitor is behind the patient, but it is advantageous to have two monitors, one on either side of the table.

While some surgeons utilise supine positioning for bilateral thoracoscopy, exposure is optimised by placing the patient in a full decubitus position, even though that requires flipping the patient between the two sides for bilateral surgery. A break in the bed helps open the intercostal spaces, while an axillary roll helps avoid nerve pressure injury.

### Trocar sites

Trocar sites are triangulated based on the expected location of the pulmonary nodules. In general, for thoracoscopy, the surgeon works from the front (anterior side) of the patient. The 5th intercostal space, anterior axillary line, typically aligns with the major fissure and is often an optimal location for the camera port. However, the camera port can be inserted anywhere along the anterior axillary line from the 5–9th inter-costal space. Care must be taken to avoid diaphragm and sub-diaphragmatic injury when entering the chest below the 6th intercostal space. The principle of triangulating the lesion with the camera port at the apex applies.

For a robotic approach, trocar placement for an anterior approach can be similarly employed. However, for robotic surgery, placement of ports in a row in the 8–9th intercostal space (just above the diaphragm) can be effective for all types of nodule locations. An additional 4th arm can be placed 2–3 rib spaces higher, in the anterior axillary line when needed.

### Surgical technique

MIS PM procedures are performed under general endotracheal anaesthesia. Single lung ventilation may be helpful, but is not mandatory since thoracic insufflation provides adequate lung collapse for surgery. Double-lumen endotracheal tubes work well in patients over 30 kg. For smaller children, single lung ventilation is more challenging, but options include bronchial blockers or selective intubation of the contralateral bronchus.

Options for wedge resection with MIS PM include bipolar energy devices (e.g., Ligasure) or surgical stapling devices. Bipolar energy devices work best for smaller superficial lesions. They may allow for a smaller cuff of normal surrounding lung tissue, but they also have an increased risk of air leak. The pleural defect can be oversewn to reduce this risk. Surgical staples are preferred for larger and deeper lesions but can also be used even for small peripheral nodules.

Steps of the procedure for MIS pulmonary wedge resection after port placement include [1] takedown of the inferior pulmonary ligament and/or opening of the anterior and/or posterior pleura as needed for mobilisation [2], inspection to identify the known lesion(s) and to assess for an occult disease in the lung or pleural space [3], application of adjunctive measures including fluorescence visualisation and/or fluoroscopy for localisation coils [4], wedge resection using bipolar energy or stapling device as above [5], inspection, possible chest tube placement and closure. Chest tubes are used selectively after isolated pulmonary wedge resection and can often be avoided for straightforward solitary wedges.

Adjunctive localisation techniques are recommended for all nodules deeper than a few mm below the pleural surface [6]. Options including hookwires, dyes, microcoils, fiducial markers, contrast media and radiotracers are well described in the literature [7, 8]. Fluorescence-guided surgery with systemically administered fluorophores, including indocyanine green [9–12] and newer agents such as pafolacianine (Cytalux), is also gaining in popularity, but is restricted in utility to nodules within about 5–8 mm of the pleural surface due to the inherent limitations in the depth of penetration of near-infrared light.

When feasible, a wedge resection is preferred over larger anatomic resections, such as lobectomy, to maintain maximum pulmonary reserve, considering there is a significant chance of additional recurrence. When anatomic resection is required, collaboration with an experienced thoracic surgeon can often permit segmentectomy rather than lobectomy. Adequate lymphadenectomy is an essential component of anatomic resections. Robotic-assisted surgery can be particularly helpful for anatomic resections.

### Tips and pitfall

- Work with an experienced staff and anaesthesiologist
- Ensure availability of appropriate materials and equipment, including but not limited to resources for one-lung ventilation, nodule localisation, MIS equipment (including fluorescence-detection if desired), appropriate energy devices and staplers and so on.
- Work with a multidisciplinary team to optimise decision-making around indications, timing and approach for PM.
- Do not be afraid to convert the surgery to open when the MIS route is difficult.
- Try to preserve as much lung parenchyma as possible while ensuring negative margins.

## Conclusion

MIS is a good option in specific paediatric pulmonary metastatic disease cases. The number and position of the lesions and the histologic type of tumour must be considered. The material and experience of the surgical team are essential factors in the decision.

## Conflicts of interest

The authors declare no conflicts of interest regarding this manuscript.

## Funding

There is no specific funding for this work.
